# The Lumbar Roll May Support Maintaining an Upright Posture of the Neck During a Prolonged Typing Task Among Individuals With Neck Symptoms: A Randomized Crossover Two-Arm Experimental Study

**DOI:** 10.7759/cureus.77179

**Published:** 2025-01-09

**Authors:** Hiroshi Takasaki, Kazuki Kikkawa, Tomoya Kitamura, Hiroki Saito

**Affiliations:** 1 Department of Physical Therapy, Saitama Prefectural University, Koshigaya-shi, JPN; 2 Center for Human Movement, Tokyo University of Technology, Tokyo, JPN; 3 Graduate Course of Health and Social Services, Saitama Prefectural University, Koshigaya-shi, JPN; 4 Physical Therapy, Tokyo University of Technology, Tokyo, JPN

**Keywords:** computer task, muscle rest, neck symptoms, rounded shoulder, typing posture

## Abstract

Background

One possible biomedical cause of neck symptoms among those who use computers is prolonged mechanical load to the peri-cervical structures, such as the forward head posture. A lumbar roll can immediately change neck posture, and thus, may be a potential tool to reduce mechanical load to the peri-cervical structures during prolonged computer tasks. Identifying the effect of such a supporting item on neck symptoms is one of the future priorities in the latest edition of a physical therapy practice guideline. This study aimed to investigate whether the lumbar roll allows the head, neck, and trunk to maintain a more upright posture and reduce loads to the peri-cervical muscles during a prolonged typing task.

Methodology

Thirty participants with neck symptoms completed a crossover randomized experiment involving two 60-min typing sessions, one with and one without a lumbar roll. Changes in craniovertebral angle (CVA), cranial cervical angle (CCA), spinal and scapular alignment, and muscle activity and changes in subjective discomfort of the neck before and after the typing task were compared.

Results

Only CVA and spinal alignment immediately improved using a lumbar roll. A notable difference in the CVA between the two conditions was seen for 58.3% of the 60-min typing task.

Conclusion

This study indicated the usefulness of the lumbar roll to maintain a more upright neck posture although its effect may or may not be meaningful. However, the lumbar roll would negligibly affect the head, trunk, and scapular postures, loads to the peri-cervical muscles, and subjective discomfort of the neck.

## Introduction

Neck symptoms including neck pain and stiff neck/shoulders are the most common work-related musculoskeletal disorders among those who use computers intensively in a seated position [1]. One possible biomedical cause of neck symptoms is prolonged mechanical load to the peri-cervical structures, such as the forward head posture (FHP) [2]. Therefore, it is necessary to identify effective strategies to minimize mechanical load to the peri-cervical structures during prolonged computer tasks through the use of supporting items to prevent the development and aggravation of neck symptoms. In fact, identifying the effect of such a supporting item on neck symptoms is one of the future priorities in the latest edition of a physical therapy practice guideline [3].

One promising item is the lumbar roll. In a seated position, anterior pelvic tilt results in reduced kyphosis of the thoracic and flexion of the lower cervical spine, and such a pelvic anterior tilt can be induced with the use of the lumbar roll. Handa et al. [4] reported in a meta-analysis that the craniovertebral angle (CVA) and cranio-cervical angle (CCA) are statistically changed to a more upright posture (i.e., a decrease of the CVA and an increase of the CCA) when wearing a lumbar roll in a sitting posture. However, this finding was based on the results of healthy individuals immediately after wearing the lumbar roll, and it is not known whether the same postural changes are observed in those with neck symptoms and whether such an upright posture is maintained during a prolonged computer task such as typing. Therefore, further research is required. Falla et al. [5] reported that the CVA decreased over time in a seated position in those with chronic neck pain more than in healthy individuals. Neck alignment is likely associated with spinal alignment as shown in the meta-analysis with the lumbar roll [4], and may be associated with the rounded shoulder as investigated in previous studies [6]. Therefore, the lumbar roll can be a low-cost supporting item enabling anyone to reduce mechanical loads on the peri-cervical structures, including head, neck, spinal, and scapular alignments and peri-cervical muscle activity during a prolonged sitting computer task.

Recently, there has been the development of the relative rest time (RRT), an electromyographic (EMG) analysis method to evaluate the dynamics of muscle activity in peri-cervical muscles during a prolonged work task. RRT is less sensitive to electrode placement and is recommended as a measure of muscle activity dynamics during prolonged low-load tasks for designs that measure on separate days [7]. RRT evaluates the percentage of time below a certain threshold in the rectified surface EMG signal. A threshold of 3 or 6 µV, which is uniform for all individuals, is recommended over the threshold with % maximum voluntary contraction or % reference voluntary contraction, which differ between individuals [8]. In particular, a threshold of 6 µV is recommended for the RRT of peri-cervical muscles during a prolonged typing task, as the standard error of measurement is often smaller than a threshold of 3 µV [9].

Therefore, the purpose of this study was to examine the effect of the lumbar for allowing the trunk and peri-cervical structures to maintain a more upright posture during a prolonged typing task, and for increasing the RRT. This study would provide biomechanical evidence for the research priority of investigating the treatment and preventative effect of a supporting item on neck symptoms [3].

## Materials and methods

Design

This study was designed as a two-arm crossover experimental study with one group using the lumbar roll and the other group not using the lumbar roll during a 60-min typing task. Measurements were taken one week apart. Written consent was submitted from each participant before data collection. The study design was approved by the institutional research ethics committee (Saitama Prefectural University, # 21091, March 31, 2022) and pre-registered in the trial registration (University Hospital Medical Information Network, UMIN000049297).

Participants

Using convenience sampling, participants were recruited via advertising at the Saitama Prefectural University from October 25, 2022, and December 31, 2022. The inclusion criteria were (1) 18-60 years of age, (2) having subjective neck symptoms with the Neck Disability Index (NDI) of >16% [10], and (3) at least 2 days per week of computer use for a total of ≥2 h per week [11]. The exclusion criteria were (1) a history of whiplash, neck fracture, or surgery, (2) inability to sit at the edge of the laboratory environment for 1 h continuously for any reason, (3) difficulty engaging in typing activities for any reason, and (4) inability to perform the standard EMG preparation for any reason (e.g., skin inflammation or trauma).

A previous meta-analysis reported an effect size of 0.77 as the difference in the CVA with and without the use of lumbar rolls [4]. Based on this data, 20 pairs were calculated to be necessary to obtain a power of 95% and a level of significance of 5% (one-sided). We decided to include 30 pairs by considering the possibility that two measurements could not be made for some cases due to the COVID-19 pandemic.

Procedures

Measurement was conducted by an author (KK). The participant sat on a chair that was adjusted to have a backrest angle of 100°, which promotes the greatest change in neck alignment with a lumbar roll [12]. The posture at the start of typing was adjusted so that the hip, knee, and elbow joints were each at 90° and the height of the monitor was adjusted to eye level.

As a baseline, participants maintained a relaxed sitting posture (i.e., resting position) with their hands on an external keyboard (TK-FCM077PBK, Elecom, Japan) for the first minute of data collection. Using Bruce’s Typing Wizard software (Roseland Productions, New York, USA), participants typed for 60 min at a comfortable pace, displaying the document to be typed. Errors were ignored. No instructions were provided with regard to the typing posture.

Measures

**Figure 1:**
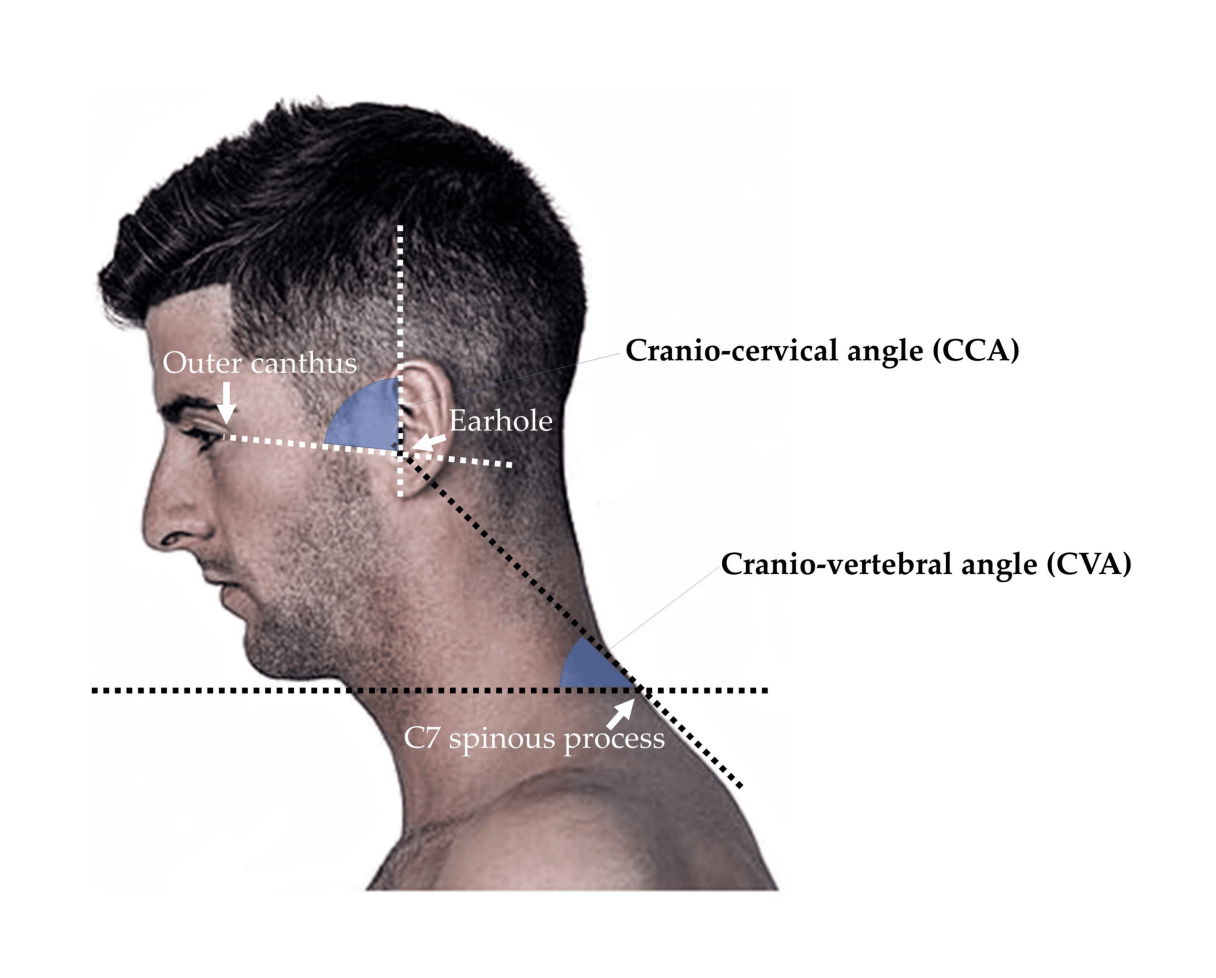
Landmarks to measure the cranio-vertebral and cranio-cervical angles. The image was created by the authors and does not belong to a participant.

The primary outcome measures included the CVA and CCA (Figure 1) during a 60-minute typing task and during a resting sitting position for 1 min before the typing task, and RRT of following seven peri-cervical muscles during the 60-min typing task; splenius capitis, upper trapezius, middle trapezius, sternocleidomastoid, serratus anterior, longissimus, and pectoralis major.

A video camera was used to capture the head and neck posture in the sagittal plane from a distance of 2 m from the side, and the image data were analyzed every minute. Image data were analyzed by one blinded author (TK) using Image J (1.46r, National Institutes of Health, Maryland, USA) after randomizing time and measurement conditions. The intra-class correlation coefficient (ICC(3,1)) (95% confidence intervals (CI)) of 50 randomly selected image data measured twice at intervals of ≥1 month by this author was 0.997 (0.994−0.998) for the CVA and 0.995 (0.990−0.997) for the CCA, respectively.

According to the protocol in a previous study [9], RRT was assessed with superficial EMG using the myoMUSCLE™ system (Noraxon USA, Inc., Arizona, USA) with a sampling frequency of 1500 Hz (digital resolution, 16bit; band-pass filter, 10Hz−500 Hz with high-pass cutoff of 10 Hz and low-pass cutoff of 500 Hz; input impedance, >100 Ω; common mode rejection ratio, >100 dB; electric gain, 200; and overall gain, 500). Briefly, self-adhesive Ag/AgCl electrodes (ECG electrodes 2009111-150, CareFusion, Finland) at a distance of 20mm between electrodes were placed on the seven peri-cervical muscles on the right side and their electrode positions as per the previous study (Appendices: Table 5) [9]. The amplitude of the muscle activity was evaluated by reducing the electrocardiogram complex, filtering the electromyography data with a 20−500 Hz band-pass filter [13,14], and calculating the root mean square with a 100 ms sliding window. RRT was calculated as the percentage of time that muscle activity was below the threshold of 6μV for more than 0.250s continuously during a 60-min typing task according to the previous studies [8,9].

Secondary outcomes included (1) spinal alignment (i.e., the angle between the posterior surface of the sacrum and the second thoracic spinous process) and scapular alignment (i.e., angle between both scapulars over the horizontal plane) during a 60-min typing task and during a resting sitting position for 1min before the typing task, and (2) bothersomeness of neck symptoms before and after the typing task using an 11-point numerical rating scale (NRS) (0=no problem, 10=extremely bothersome). Other characteristics of the participants were also obtained: age, sex assigned at birth, height, weight, subjective dominant hand side (left, right, or both), location of symptoms on an upper body chart [15,16], the NDI, duration of symptoms (<7 days, 8 days−3 months, or >3 months), Katakori Disability Index (KDI) for neck symptom severity [17], the 4-Item pain intensity measure (P4) for pain intensity [18,19], and EuroQol-5Dimensions (EQ-5D) for quality of life [20].

Regarding the spinal and scapular alignments, three-dimensional accelerometers (Myomotion, Noraxson, Arizona, USA) were fixed with surgical tape over the left and right acromion, posterior pelvis, and second thoracic spinous process, and angle data were collected by synchronizing with an EMG at a sampling frequency of 100Hz. Data in which the angle between both scapulars on the horizontal plane changed by more than 100° in 0.1s were excluded as outliers. The average value of every minute was calculated from the resting state. A smaller value for the sagittal spinal alignment indicated a more slouching posture and a smaller value for the scapular alignment indicated a more rolled shoulder posture.

The NDI is a patient-reported outcome measure (PROM) developed to assess disability in individuals with neck pain and has been widely used to assess disability associated with neck pain. Notably, a reliable and valid Japanese version of this index had been developed in 2006 [21]. The NDI includes 10 items, with higher % scores indicating greater disability due to neck pain. Considering that the Japanese version set a cutoff of 15% for the degree of disability sufficient to require a visit to a medical facility [10], we adopted a cutoff value of ≥16% as our inclusion criterion.

The KDI is a PROM developed to assess disability due to stiff neck/shoulders, that is nonspecific symptoms such as discomfort or dull pain due to muscle stiffness around the occiput, cervical spine, acromion, and scapular area [17,22,23]. The KDI includes 11 items, with higher mean scores (0−14) indicating greater disability due to stiff neck/shoulders. Notably, content validity and unidimensional internal structure have been identified in the Japanese population [17,22,23].

The P4 is a PROM consisting of four 11-point NRSs that assess average pain intensity throughout the morning, noon, and evening over the past 2 days and pain intensity during activity. The P4 is a reliable and responsive measure of pain intensity [18,19], with higher mean scores (0−10) indicating greater pain intensity. Minimum clinically important difference (MCID) was reported to be 1.64 [19].

The EQ-5D is a PROM that assesses the quality of life and consists of five items and five statements regarding quality of life [20]. A Japanese version of the questionnaire and scoring method was developed in 2015 [24]. Higher scores (0−1) indicate a better quality of life [25].

Randomization

An author (HT) assigned the order of measurement for the two groups based on a computerized random table. The lumbar roll (The Original McKenzie® Lumbar Roll™, OPTP, USA) was placed at the L4 level after the participants were seated deeply in the chair.

Analysis

For the primary outcomes of the CVA and CCA, the means of the two experimental groups in the resting position for 1 min immediately before typing were compared with a paired one-tail t-test. Further, the CVA, CCA, and RRT means of the two experimental groups over the 60-min typing task were analyzed with treatment effect estimation with the use of 95% CIs of the mean differences between the two measurements with and without the lumbar roll against the minimum detectable changes (MDCs) or MCIDs, as recommended in physical therapy research [26]. The effect of the use of the lumbar roll was interpreted based on the nine categories as proposed by a previous study [27]. Briefly, if the 95% CIs of the mean of the difference between the two measurement crosses the MCID and the lower 95% CI is more than 0, the intervention is interpreted as being effective but may or may not be meaningful. If the 95% CI is between 0 and MCID, the intervention is interpreted as being effective, but the effect is too small to be meaningful. If the 95% CIs crosses 0, is close to 0, and the upper 95% CI is less than the MCID, the intervention is interpreted as having no effect, is trivially effective, or is trivially harmful. Regarding the CVA, Fall et al. [5] reported that the CVA for longer sitting posture significantly increased in those with neck pain compared to healthy individuals, and the upper limit of the 95% CI for the increase in the CVA for healthy individuals was 3.4°. Therefore, the MCID for the CVA was set at 3.5° in this study. Regarding the CCA, a systematic review [28] reported that the mean pooled difference in the CVA between those with and without neck pain was 2.93° (95% CIs, 0.91°−4.95°). Therefore, the MCID for the CVA was set at 5° in this study. Regarding the RRT, reported MDCs of the seven peri-cervical muscles during the 60-min typing task [9] were used in this study.

For the secondary outcomes of the spinal and scapular alignments, the means of the two experimental groups in the resting position for 1 min immediately before the typing task was compared with a paired two-tail t-test, and the means of the two experimental groups during the 60-min typing task were analyzed with the one-dimensional Statistical Parametric Mapping (SPM) (spm1d v0.4.7 for MATLAB) [29], which is an analysis method to detect statistically significant differences across time series and spatial data. Between-group changes in bothersomeness of neck symptoms before and after the typing task were compared in a one-way repeated measures analysis of variance. The NDI, KDI, P4, EQ-5D, and bothersomeness of neck symptoms immediately before and after the typing task were compared between the two measurement days and between the two experimental groups with the paired two-tail t-test. In addition, equivalence between the two measurement days was investigated whether any participant exceeded the MCID of 1.64 with the P4 that has acceptable responsiveness [19].

Other than for the SPM, all statistical analyses were performed using IBM SPSS version 25 (IBM Corp, Armonk, New York, USA). Statistical significance was set at 5%. The effect size for each statistical test was calculated by Cohen’s d, and the effect size for the SPM was the mean of the effect sizes over the entire continuous interval with statistically significant differences. The interpretations of the effect size were as follows: small at 0.2, moderate at 0.5, and large at 0.8.

## Results

**Figure 2:**
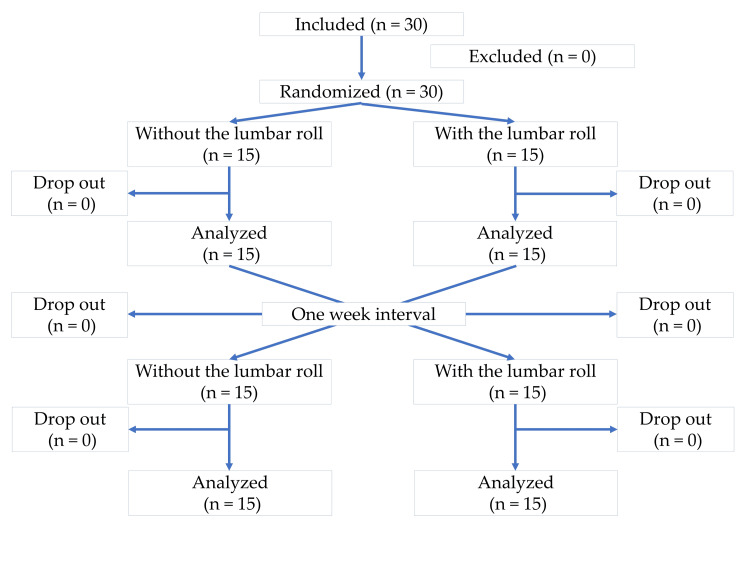
A flowchart of the participants. The image was created by the authors of this article.

**Figure 3:**
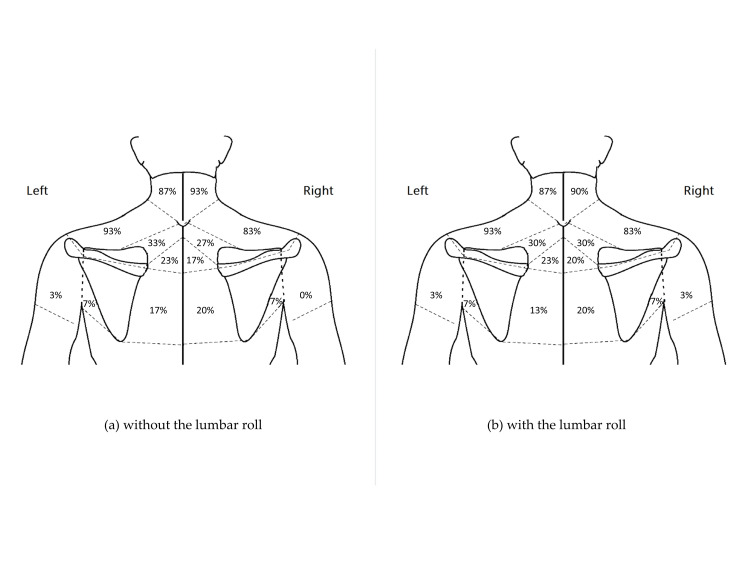
Symptom locations before the typing task in the 30 participants. The values are expressed as a percentage of the 30 participants. The image was created by the authors of this article.

Thirty participants (8 men and 22 women) completed the two measurements (Figure 2), whose mean (SD) age and body mass index were 22.9 (7.2) years and 20.8 (3.3) kg/m^2^, respectively. Regarding the duration of symptoms, 8 patients had symptoms for 8 days to 3 months, and 22 patients had symptoms for >3 months. Symptom locations are summarized in Figure 3. All participants were right-handed. There were no statistically significant differences in the NDI, KDI, P4, EQ-5D, and bothersomeness of neck symptoms immediately before and after the typing task on either of the two measurement days (Table 1) or for the two experimental groups (Table 2). There was nobody whose P4 difference between the two measurement days exceeded the minimal clinically important difference (MCID). No one could type the one sentence displayed without looking at their hand.

**Table 1:** Subjective status at the two measurement days. NDI: Neck Disability Index; KDI: Katakori Disability Index; P4: 4-Item Pain Intensity Measure; EQ-5D: EuroQol-5Dimensions Values are presented with mean ± SD. *One week after the initial session ^†^Two-tail t-test ^‡^Absolute value

Variable	Initial session	Second session*	P-value^†^	Effect size (Cohen’s d)^ ‡^
NDI (%)	27.0 ± 12.7	24.9 ± 8.7	0.087	0.32
KDI (0−14)	7.0 ± 2.5	6.9 ± 2.8	0.857	0.03
P4 (0−10)	3.8 ± 1.6	3.7 ± 1.3	0.279	0.20
EQ-5D (0−1)	0.66 ± 0.11	0.67 ± 0.13	0.799	0.05
Neck discomfort immediately before typing (0−10)	2.8 ± 1.4	3.0 ± 1.4	0.420	0.15
Neck discomfort immediately after typing (0−10)	5.5 ± 1.7	5.3 ± 1.5	0.385	0.16

**Table 2:** Subjective status in the two intervention conditions. NDI: Neck Disability Index; KDI: Katakori Disability Index; P4: 4-Item Pain Intensity Measure; EQ-5D: EuroQol-5Dimensions. Values are presented with mean ± SD. *Two-tail t-test ^†^Absolute value

Variable	Without the lumbar roll	With the lumbar roll	P-value*	Effect size (Cohen’s d)^†^
NDI (%)	24.7 ± 9.1	27.1 ± 12.4	0.053	0.37
KDI (0–14)	7.0 ± 2.5	6.9 ± 2.7	0.785	0.05
P4 (0–10)	3.8 ± 1.6	3.7 ± 1.4	0.721	0.07
EQ-5D (0–1)	0.66 ± 0.12	0.67 ± 0.13	0.281	0.20
Neck discomfort immediately before typing (0–10)	2.8 ± 1.5	3.0 ± 1.3	0.420	0.15
Neck discomfort immediately after typing (0–10)	5.5 ± 1.7	5.3 ± 1.5	0.564	0.11

**Figure 4:**
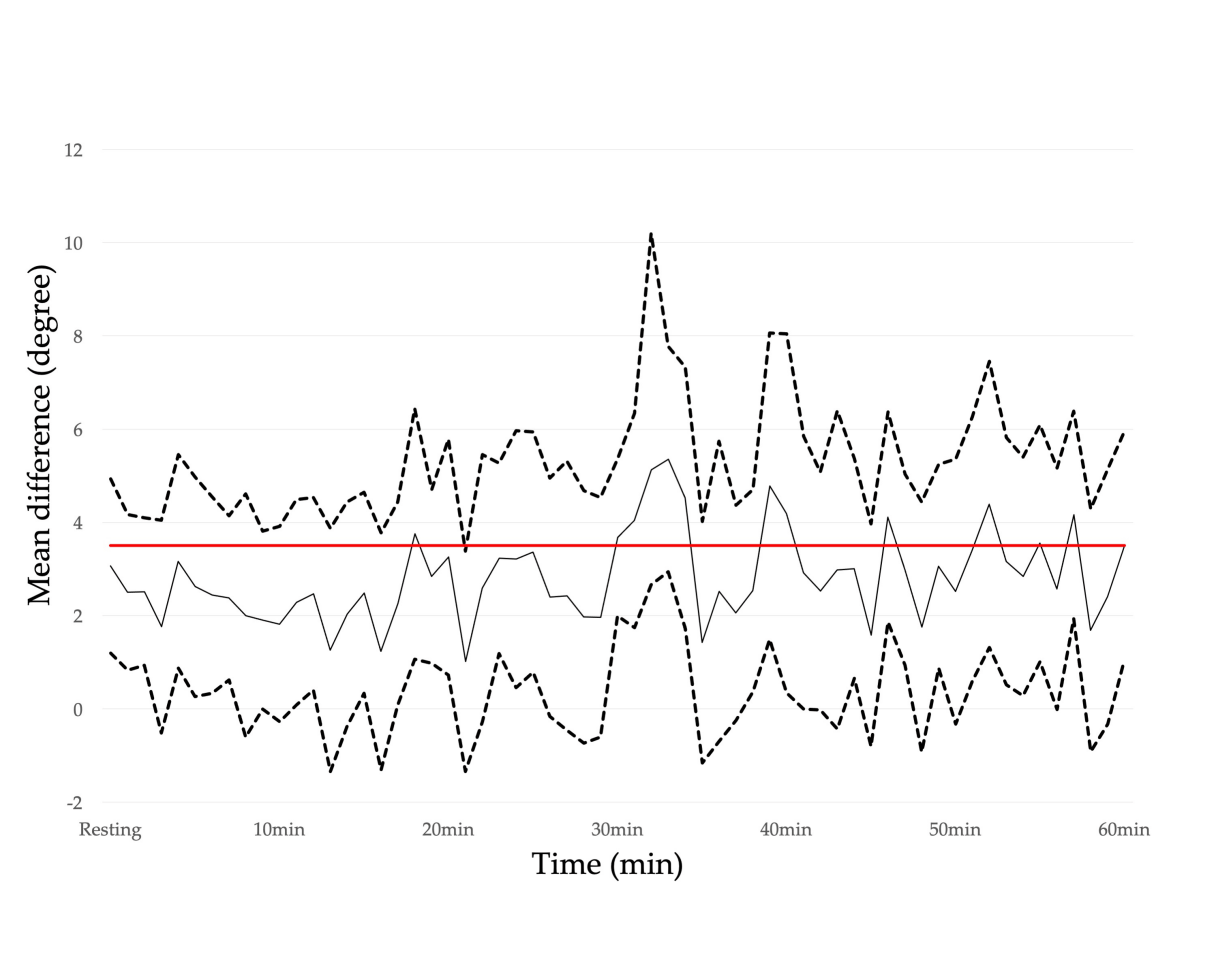
Temporal variations of the mean difference in the craniovertebral angle between the two conditions with and without the lumbar roll. The dotted lines represent the 95% confidence intervals and the solid black line represents the mean difference. The red line represents a threshold to interpret the effect of an intervention. Mean difference: positive values indicate a greater craniovertebral angle with the lumbar roll than without the lumbar roll (i.e., a more upright posture). The image was created by the authors of this article.

**Figure 5:**
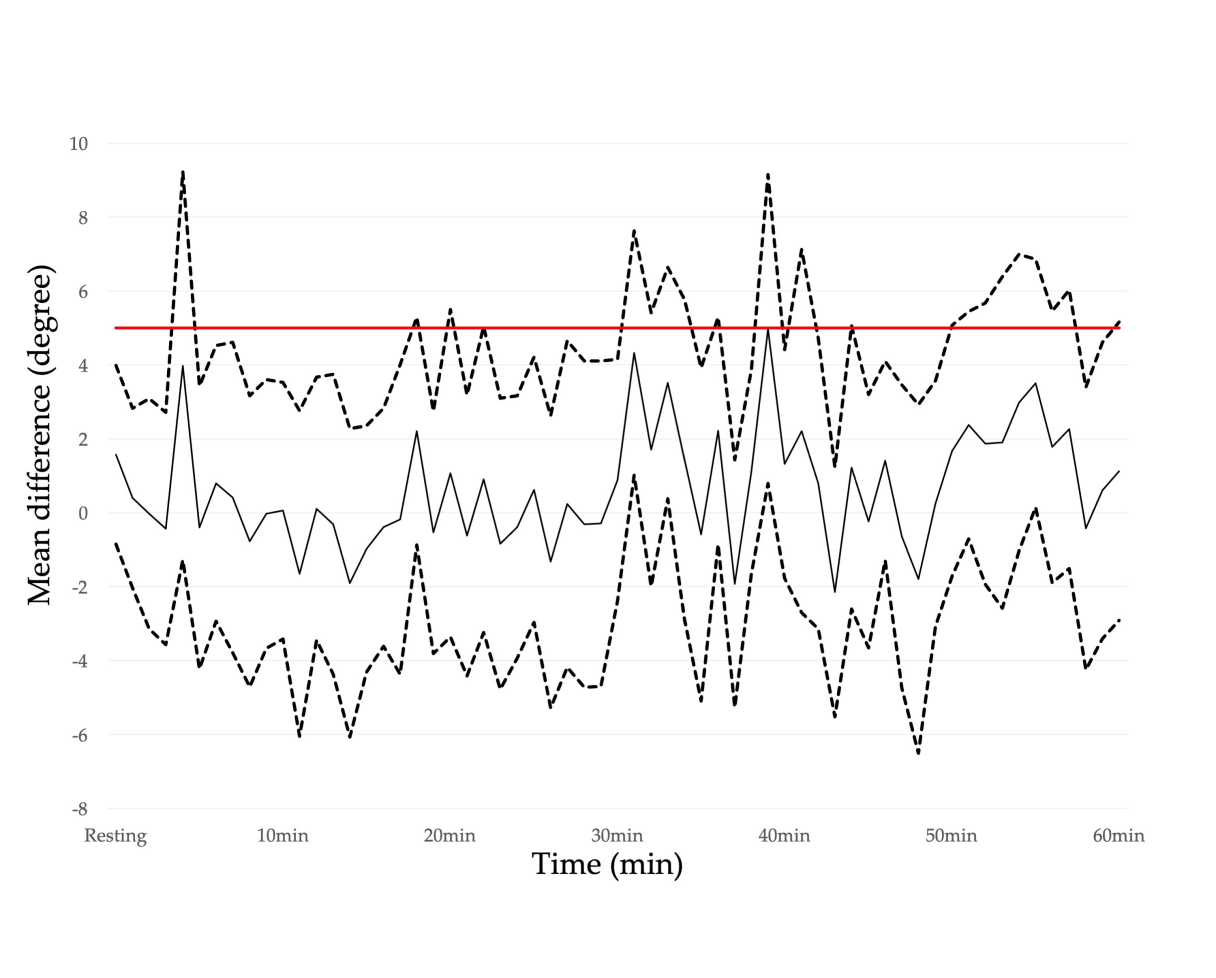
Temporal variations of the mean difference in the cranio-cervical angle between the two conditions with and without the lumbar roll. The dotted lines present the 95% confidence intervals and the solid line represents the mean difference. The red line represents a threshold to interpret the effect of an intervention. Mean difference: positive values indicate a smaller cranio-cervical angle with the lumbar roll than without the lumbar roll (i.e., a more upright posture). The image was created by the authors of this article.

**Figure 6:**
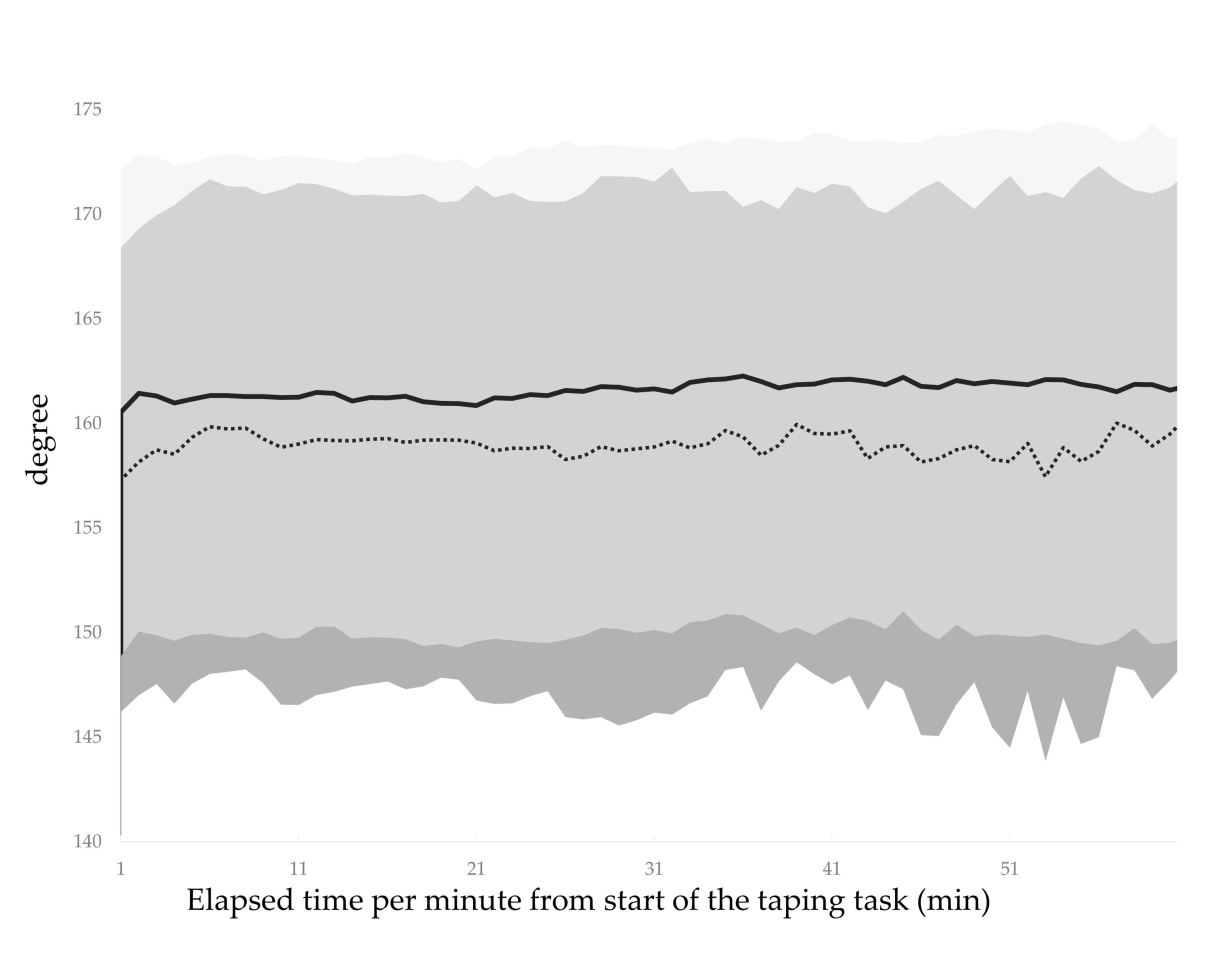
Temporal variations of the scapular alignment for two conditions with and without the lumbar roll. The dotted line presents the value without the lumbar roll and the solid line presents the value with the lumbar roll. A greater value indicates a posture with less rounded shoulder. Shades represent standard deviations. The image was created by the authors of this article.

**Figure 7:**
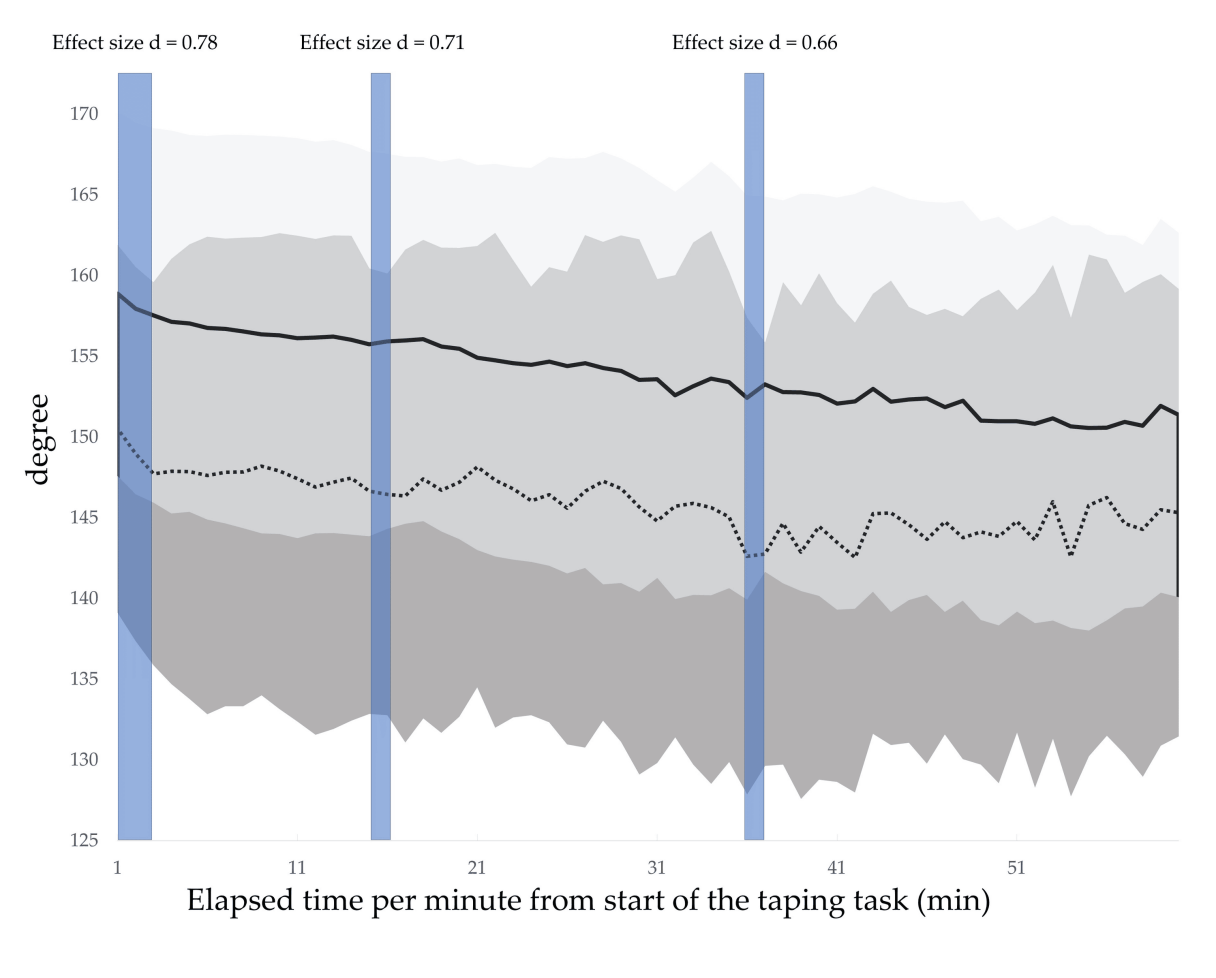
Temporal variations of the spinal alignment for two conditions with and without the lumbar roll. The dotted line represents the value without the lumbar roll and the solid line represents the value with the lumbar roll. A greater value indicates a more upright posture. Shades represent standard deviations. Blue shade indicates the time zone with a statistically significant difference between the two conditions in the one-dimensional Statistical Parametric Mapping. The image was created by the authors of this article.

**Figure 8:**
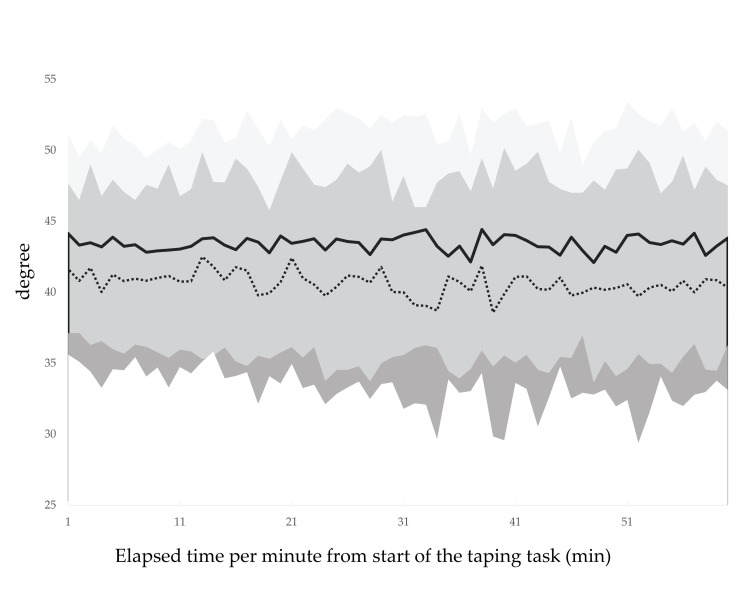
Temporal variations of the cranio-vertebral angles for the two conditions with and without the lumbar roll. The dot line presents the value without the lumbar roll and the solid line presents the value with the lumbar roll. A greater value indicates a more upright posture. Shades present standard deviations. The image was created by the authors of this article.

**Figure 9:**
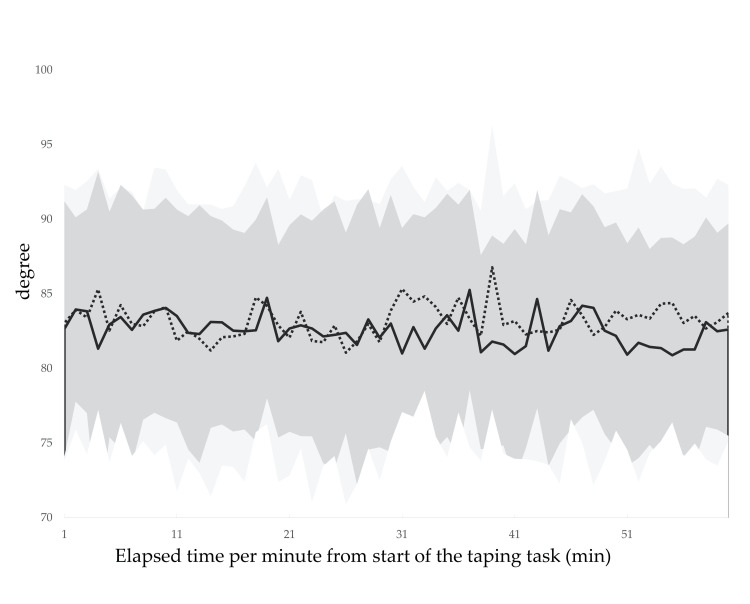
Temporal variations of the cranio-cervical angles for the two conditions with and without the lumbar roll. The dotted line presents the value without the lumbar roll and the solid line presents the value with the lumbar roll. A greater value indicates a more upright posture. Shades present standard deviations. The image was created by the authors of this article.

The CVA, CCA, and spinal and scapular alignments in the resting position with and without lumbar roll are shown in Table 3, where moderate effect sizes for the CVA and spinal alignment indicate that the use of the lumbar roll results in a more upright posture. Figures 4-5 show the temporal variations of the mean difference in the CVA and CCA. Regarding the CVA, the interpretation of the effect with the use of the lumbar roll to maintain a more upright posture during a prolonged typing task was that the lumbar roll is effective but may or may not be meaningful for 58.3% of the 60-min typing task. Regarding the CCA, the interpretation of the effect was that the lumbar roll has no effect, is trivially effective, or is trivially harmful for 65.0% of the 60-min typing task. There were no obvious alignment changes observed over time for either the CVA or the CCA (Appendices: Figures 8-9). Figures 6-7 show the temporal variation of the spinal and scapular alignments, respectively. In SPM, only three intervals of spinal alignment showed statistically significant differences (all P<0.05) between the two experimental groups (Figure 7). Regarding the RRT, most interpretations of the effect of the use of the lumbar roll to increase the RRT were that the lumbar roll has no effect, is trivially effective, or is trivially harmful (Table 4). Regarding the bothersomeness of neck symptoms immediately before and after the typing task, there was no statistically significant interaction between the two experimental groups (P=0.286).

**Table 3:** The alignment measures at the resting position immediately before the typing task. CVA: craniovertebral angle; CCA: cranio-cervical angle Values are presented with mean ± SD. *One-tail t-test ^†^Two-tail t-test ^‡^Absolute value

Variable (Degree)	Without the lumbar roll	With the lumbar roll	P-value	Effect size (Cohen’s d)^‡^
CVA*	45.3 ± 7.0	48.4 ± 7.1	0.001	0.61
CCA*	77.5 ± 6.8	75.9 ± 5.8	0.098	0.24
Spinal alignment^†^	152.3 ± 10.9	160.8 ± 11.4	0.004	0.57
Scapular alignment^†^	157.3 ± 11.1	160.5 ± 11.7	0.267	0.21

**Table 4:** The relative rest time (RRT) during a 60-min typing task with and without the lumbar roll. MDC: minimum detectable change

Muscle	Without the lumbar roll (A)	With the lumbar roll (B)	Difference of (B)-(A) (mean (95% CIs))	RRT MDC	Interpretation of the effect with the use of the lumbar roll to increase the RRT
Splenius capitis	99.9 ± 0.4	100.0 ± 0.1	0.1 (0.0 to 0.3)	26.4	The lumbar roll is effective, but the effect is too small to be meaningful.
Upper trapezius	84.7 ± 22.0	90.4 ± 17.5	5.7 (-1.2 to 12.7)	52.1	The lumbar roll has no effect, is trivially effective, or is trivially harmful.
Middle trapezius	99.0 ± 2.7	99.9 ± 0.2	0.8 (-0.2 to 1.9)	6.4	The lumbar roll has no effect, is trivially effective, or is trivially harmful.
Sternocleidomastoid	99.9 ± 0.2	99.8 ± 0.3	-0.1 (-0.2 to 0.1)	1.1	The lumbar roll has no effect, is trivially effective, or is trivially harmful.
Serratus anterior	96.3 ± 15.7	99.9 ± 0.2	3.7 (-2.2 to 9.5)	32.3	The lumbar roll has no effect, is trivially effective, or is trivially harmful.
Longissimus	99.3 ± 2.1	99.9 ± 0.3	0.6 (-0.2 to 1.4)	9.2	The lumbar roll has no effect, is trivially effective, or is trivially harmful.
Pectoralis major	98.9 ± 2.7	98.2 ± 8.8	-0.7 (-4.2 to 2.8)	11.8	The lumbar roll has no effect, is trivially effective, or is trivially harmful.

## Discussion

Immediate effects on the craniovertebral angle (CVA), cranio-cervical angle (CCA), and spinal alignment

Immediately after installing the lumbar roll, CVA and spinal alignment showed a more upright posture than the control group without the lumbar roll, whose effect size was moderate. On the other hand, there was no statistically significant change in the CCA and scapula alignment. The previous meta-analysis [4] showed that the CVA increased and the CCA decreased immediately after installing the lumbar roll, but in this study, only the CVA matched the results of the meta-analysis [4]. Considering that the participants in this meta-analysis [4] were healthy individuals and the participants in this study were symptomatic individuals, the reason why CCA did not change with the lumbar roll could possibly be related to some characteristics of symptomatic individuals, such as limited range of motion of the neck or abnormal relative flexibility of the peri-cervical muscles, for example. Since the influence of the lumbar roll on alignment is expected to diminish in the order of lumbar spine, trunk, CVA, and CCA, it is likely that the influence of some characteristics of those with neck symptoms would have been superior to the indirect influence of the lumbar roll on the CCA. The clinical implications of this finding may suggest that simple postural instruction using the lumbar roll is not sufficient for patients with neck symptoms who need to correct their upper cervical alignment and that therapeutic interventions, including hands-on techniques for the upper cervical spine, may be required.

The CVA and spinal alignment over time

The CVA and spinal alignment before the typing task showed an upright posture with the lumbar roll, but they began to change differently once the typing task started. Regarding the CVA, the interpretation of the effect with the use of the lumbar roll to maintain a more neutral alignment during the prolonged typing task was that the lumbar roll is effective but may or may not be meaningful for 58.3% of the 60-min typing task. As shown in Figure 2, there is no clear shift indicating a difference between the two groups over time. Looking at the CVA over time for each group, there was no obvious change over time. On the other hand, spinal alignment showed differences between the two groups just after the start of the typing task, and only in two other intervals lasting a few minutes. There is a tendency for the angle of spinal alignment to decrease over time with or without lumbar, but the degree of decrease seems to average about 5°, and it is questionable whether this has any clinical effect given that the CVA shows no change over time.

A decrease in the CVA implies an increase in the flexion moment load on the lower cervical spine. In fact, there are many who improve their neck symptoms with lower cervical extension which reduces the flexion load on the lower cervical spine [15,30]. Therefore, lumbar rolls may be beneficial as a supporting tool to reduce lower cervical flexion load during prolonged computer tasks, especially for those with neck symptoms that are exacerbated by lower cervical flexion load. In addition, except for those with low back pain whose symptoms are strongly influenced by trunk alignment, the impact of 60-min of typing activity, with or without lumbar roll, on trunk alignment is limited. Therefore, the excessive advice about posture to frequently re-correct posture earlier than 60-min seems not to be supported by this finding. Rather, the data in this study may serve to reassure patients who are excessively anxious about their posture at work.

The CCA and scapula alignment over time

During the typing task, the CCA and scapula alignment showed little difference with and without lumbar roll and no obvious change over time. One possible reason for the minimal difference in the CCA was that all participants had to type while looking at their hands, which caused the CCA to fluctuate repeatedly. Regarding the scapula alignment, a previous study reported no correlation between scapula alignment in the supine position and FHP in the sitting or standing position [6]. Although the present study measured the scapula alignment in the sitting position, the changes in the scapula alignment over time differed from that of the CVA, suggesting that the results could be interpreted as similar to the findings of the previous study [6].

Relative rest time (RRTs) of the peri-cervical muscles and the subjective discomfort

This study evaluated the RRT as one of the effects of the lumbar roll on load experienced by the seven peri-cervical muscles. The results indicated that the use of lumbar rolls did not reduce the load on the peri-cervical muscles, or if it did, it was too small to be meaningful. This result is consistent with the lack of scapular alignment changes with the lumbar roll. As a preliminary finding of the clinical effects of the lumbar roll on the patients with neck symptoms, this study also compared the change in the 11-point NRS on neck discomfort with and without the lumbar roll, but no statistically significant interaction was found. The clinical implication of these results may indicate that the use of the lumbar roll to reduce muscle fatigue and discomfort around the neck cannot be expected to be effective when those with neck symptoms are considered to be a homogeneous group. Moreover, lumbar roll use alone is not thought to contribute greatly to the prevention of the onset of cervical symptoms. With regard to future validation of the effect of support tools in reducing/preventing neck symptoms, a different support tool than the lumbar roll would be investigated.

Study limitations

First, this study did not divide the participants into certain subgroups, including sex/gender. As mentioned above, it is possible that the results when using the lumbar roll may differ owing to some physical characteristics such as limited range of motion. This study showed the effect of the lumbar roll when viewed as a homogeneous group of neck symptoms, which is a limitation of the study, and further validation is needed regarding the effect of the lumbar roll in certain clinical subgroups. Second, this study evaluated neck discomfort on an 11-point NRS as a preliminary investigation into the use of the lumbar roll to reduce neck symptoms and prevent the development of those. However, the reliability, validity, and responsiveness of this PROM have not been verified, which is a limitation of the study, and caution was warranted in interpreting the numerical values. However, because this study utilized a crossover design, differences in perceived discomfort between participants were balanced out, and there was no interaction in the difference between pre- and post-typing tasks, the subjective symptom reduction effect of using the lumbar roll is probably negligible.

## Conclusions

This study found that the lumbar roll can help us maintain a more upright neck posture, but its effect may or may not be meaningful during prolonged typing tasks. However, in terms of the head, spinal and scapular postures, and load to the peri-cervical muscles, the effect of using the lumbar roll does not seem to be clinically meaningful.

## Appendices

**Table 5:** Electrode locations

Muscles (Right side)	
Splenius capitis muscle	2 cm right lateral from the spinous process of the 4th cervical vertebrae electrode on the cephalic side.
Upper trapezius muscle	Medial electrode midway between the spinous process of the 7th cervical vertebra and the right acromion.
Middle trapezius muscle	2 cm right lateral from the spinous process of the 4th cervical vertebrae electrode on the cephalic side.
Sternocleidomastoid muscle	Located halfway between the mastoid process and the sternal notch, running parallel to the muscle fibers.
Serratus anterior muscle	Horizontally just below the axillary area, at the level of the inferior tip of the scapula, and just medial of the latissimus dorsi.
Longissimus muscle	2 cm right lateral from the third lumbar spinous process is the cephalad electrode.
Pectoralis major muscle	2 cm inside from the right axilla is the outer electrode.
